# Supplementary material for: Rawsamble: overlapping raw nanopore signals using a hash-based seeding mechanism
Source: Bioinformatics. 2026 Feb 26;42(3):btag087. doi: 10.1093/bioinformatics/btag087 (PMC12975284; doi:10.1093/bioinformatics/btag087)
Supplement: btag087_Supplementary_Data [file btag087_supplementary_data.pdf]

Supplementary Material for Rawsamble: Overlapping Raw Nanopore Signals using a Hash-based Seeding Mechanism

A. RawHash2 Overview

Rawsamble builds improvements over RawHash2 (Firtina et al., 2023, 2024), a mechanism that provides a hash-based similarity identification between a raw signal and a reference genome. We show the overview of RawHash2 in Supplementary Figure S1. RawHash2 has four key steps.

First, to generate sequences of signals that can be compared to each other, RawHash2 generates signals of k-mers, called *events*, from both a reference genome and raw signals. To generate events from reference genomes, it uses a lookup table, called *k-mer model*, that provides the expected signal value (i.e., event value) as a floating value for each possible k-mer where k is usually 6 or 9, depending on the flow cell version. To identify events (i.e., k-mers) in raw signals, RawHash2 performs a segmentation technique to detect the abrupt changes in signals, which enables identifying the regions in signals generated when sequencing a particular k-mer. RawHash2 uses the average value of signals within the same region as an event value after identifying outliers within the region. Due to the variations, oversegmentation issues used in the classical segmentation algorithms (e.g., t-test), and noise in nanopore sequencing, event values generated from the same k-mer can slightly differ from each other, making it challenging to directly match the event values to each other to identify matching k-mers between a reference genome and raw signals. These variations are usually handled by performing homopolymer compression (HPC) type strategies, where consecutive signals with similar values are collapsed into a single signal (Zhang et al., 2021; Firtina et al., 2023, 2024; Shivakumar et al., 2024).

Second, to further mitigate this noise issue in events, RawHash2 quantizes the event values such that slightly different event values can be quantized into the same value (i.e., bucketing) to enable direct matching of quantized event values between a reference genome and raw signals. To enable an accurate quantization, RawHash2 identifies the range of values that are assigned to the same quantized value dynamically according to the nanopore model.

Third, to reduce the number of potential matches without reducing accuracy, RawHash2 concatenates the quantized event values of consecutive events (i.e., consecutive k-mers) and generates a hash value from these concatenated values.

Fourth, for the reference genome, these hash values are stored in a hash table along with their position information, which is usually known as the indexing step in read mapping. RawHash2 uses the hash values of raw signals to query the previously constructed hash table to identify matching hash values, known as *seed hits*, between a reference genome and a raw signal. Seed hits are used to identify chains of seed matches within proximity using the chaining algorithm proposed in minimap2 (Li, 2018). RawHash2 identifies the best chain among many chains based on a weighted decision strategy that takes several metrics into account, such as chaining score and mapping quality. RawHash2 uses the best chain as the best mapping for a read and reports a single mapping per read.

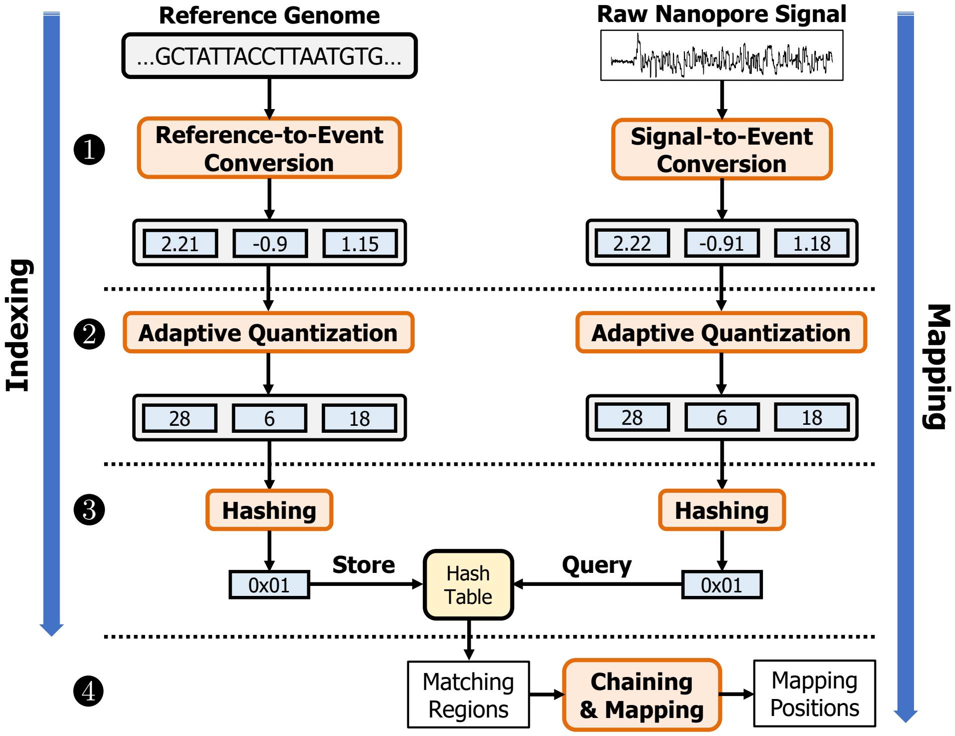

Figure S1 Overview of RawHash2.

B. Estimating the AWS Cloud Computing Costs

Supplementary Table S1 shows the estimated cloud computing prices when using the corresponding instances provided by Amazon Web Services (AWS). We estimate AWS compute costs for each tool by selecting the cheapest EC2 instance in the `us-east-1` region such that 1) the architecture is `x86_64`, 2) the vCPU count is fixed to 64 to match our experimental configuration, and 3) the **memory** is at least the measured peak memory reported in Table 3. For GPU basecalling, we additionally require a single GPU with 48 GB VRAM.

To compute a *compute-only* monetary cost estimate, we multiply the instance on-demand hourly rate as provided by AWS[2] by the measured wall-clock elapsed time. We report the ratio of each tool's estimated cost relative to Rawsamble on the same dataset (values in parentheses). We highlight rows where Rawsamble is cheaper or more expensive. This estimate excludes storage and data transfer charges, and prices are specific to the selected region and the retrieval date.

Table S1 Estimated monetary costs when using AWS cloud computing to execute each workload.

Organism	Tool	AWS instance (64 vCPU, x86_64)	Rate (USD/hr)	Elapsed (hh:mm:ss)	Est. cost (USD)
D1	**Rawsamble**	`c6a.16xlarge`	2.4480	0:52:07	2.13
E. coli	**Dorado CPU (Fast) + Minimap2**	`c6a.16xlarge`	2.4480	2:56:45	7.21 (3.39×)
	**Dorado CPU (HAC) + Minimap2**	`c6a.16xlarge`	2.4480	13:31:19	33.10 (15.57×)
	**Dorado GPU (HAC) + Minimap2**	`g6e.16xlarge`	7.5772	0:26:48	3.38 (1.59×)
D2	**Rawsamble**	`c6a.16xlarge`	2.4480	0:01:21	0.06
Yeast	**Dorado CPU (Fast) + Minimap2**	`m5a.16xlarge`	2.7520	0:31:11	1.43 (25.97×)
	**Dorado CPU (HAC) + Minimap2**	`r6a.16xlarge`	3.6288	2:30:28	9.10 (165.22×)
	**Dorado GPU (HAC) + Minimap2**	`g6e.16xlarge`	7.5772	0:02:00	0.25 (4.59×)
D3	**Rawsamble**	`c6a.16xlarge`	2.4480	0:06:20	0.26
Green Algae	**Dorado CPU (Fast) + Minimap2**	`c6a.16xlarge`	2.4480	0:30:17	1.24 (4.78×)
	**Dorado CPU (HAC) + Minimap2**	`c6a.16xlarge`	2.4480	7:29:20	18.33 (70.95×)
	**Dorado GPU (HAC) + Minimap2**	`g6e.16xlarge`	7.5772	0:03:04	0.39 (1.50×)
D4	**Rawsamble**	`c6a.16xlarge`	2.4480	0:45:08	1.84
Human	**Minimap2 + Dorado CPU (Fast)**	`c6a.16xlarge`	2.4480	5:54:27	14.46 (7.85×)
	**Minimap2 + Dorado CPU (HAC)**	`m5a.16xlarge`	2.7520	18:45:18	51.61 (28.03×)
	**Minimap2 + Dorado GPU (HAC)**	`g6e.16xlarge`	7.5772	0:13:48	1.74 (0.95×)
D5	**Rawsamble**	`c6a.16xlarge`	2.4480	16:04:39	39.36
E. coli (R10.4.1)	**Dorado CPU (Fast) + Minimap2**	`c6a.16xlarge`	2.4480	17:16:52	42.30 (1.07×)
	**Dorado CPU (HAC) + Minimap2**	`c6a.16xlarge`	2.4480	135:05:47	330.72 (8.40×)
	**Dorado GPU (HAC) + Minimap2**	`g6e.16xlarge`	7.5772	4:59:49	37.86 (0.96×)

[2] We use the online calculator at `https://calculator.aws/#/createCalculator/ec2-enhancement`

C. Generating a Gold Standard Assembly

To generate a gold standard assembly using R9.4 Simplex reads, we explore two approaches.

First, we use the raw basecalled sequences directly with a set of state-of-the-art assemblers identified from recent benchmarks (Sun et al., 2021; Wick and Holt, 2021; Cosma et al., 2022; Yu et al., 2024), namely Hifiasm (Cheng et al., 2021), Verkko (Rautiainen et al., 2023), LJA (Bankevich et al., 2022), HiCanu (Nurk et al., 2020), and Flye (Kolmogorov et al., 2019).

Second, we apply error correction to the reads using the HERRO tool (Stanojevic et al., 2024) developed by Oxford Nanopore Technologies (ONT), which is also integrated into the latest versions of Dorado as *dorado correct*. HERRO corrects erroneous R9.4 and R10.4 data, enabling their use as a replacement for accurate PacBio HiFi reads required by state-of-the-art hybrid assembly approaches. Supplementary Table S2 shows the estimated sequencing depth of coverage before and after the HERRO correction for each dataset.

For the high-coverage D1 *E. coli* dataset, Hifiasm outputs a fragmented assembly, while Verkko requires extensive parameter tuning (specifically in terms of coverage, `--unitig-abundance`, and `--base-k`) to achieve desirable contiguity and completeness. LJA produces an almost perfect assembly when a minimum length filter of 30kbp is applied to the corrected reads, as suggested by the HERRO paper. Flye performs comparably to LJA without the need for error correction. It should be noted that the golden standard assembly for D5 *E. coli* dataset was limited in terms of assembly coverage, using `--asm-coverage 50`, as Flye raised an error regarding an otherwise too high coverage level.

For the other datasets, with or without error correction, most assemblers either fail to produce assemblies or generate suboptimal results, except for Flye. We attribute this to their sensitivity to inaccuracies in the uncorrected reads and the reduced coverage after correction. Supplementary Table S2 shows how the coverage levels change after correction for each dataset. Notably, Flye works well (and sometimes even slightly better) with uncorrected reads, highlighting its robustness to noisy ONT reads.

Based on these observations, we select Flye as the assembler to generate the gold standard assemblies in our evaluations, given its ability to handle noisy ONT reads without the need for error correction and its consistent performance across different datasets. Therefore, we use Flye to construct the gold standard assemblies from the basecalled reads in our study.

Table S2 Coverage levels before and after error correction for each dataset.

Dataset	Coverage Before Correction	Coverage After Correction
D1 *E. coli*	445×	240×
D2 *Yeast*	32×	12×
D3 *Green algae*	5.6×	3.7×
D4 *Human*	0.6×	0.002×
D5 *E. coli* (R10.4.1)	1796.9×	338×

D. Configuration

D.1. Parameters

In Supplementary Table S3, we show the parameters of each tool for each dataset. In Supplementary Table S4, we show the details of the preset values that Rawsamble sets in Supplementary Table S3. For minimap2 (Li, 2018), we use the same parameter setting for all datasets. For miniasm (Li, 2016), we use the default parameter settings for all datasets.

Table S3 Parameters we use in our evaluation for each tool and dataset in mapping.

Tool	D1 *E. coli*	D2 *Yeast*	D3 *Green Algae*	D4 *Human*	D5 *E. coli (R10.4.1)*
Rawsamble	-x ava -t 64	-x ava -t 64	-x ava -t 64	-x ava -t 64	-x ava –r10 -t 64
Minimap2			-x ava-ont –for-only -t 64		
Dorado CPU (Fast)			basecaller -x cpu fast		
Dorado CPU (HAC)			basecaller -x cpu hac		
Dorado GPU (HAC)			basecaller hac		

Table S4 Corresponding parameters of presets (-x) in Rawsamble.

Preset	Corresponding parameters	Usage
ava-viral	-e 6 -q 4 -w 0 –sig-diff 0.45 –fine-range 0.4 –min-score 20 –min-score2 30 –min-anchors 5 –min-mapq 5 –bw 1000 –max-target-gap 2500 –max-query-gap 2500 –chain-gap-scale 1.2 –chain-skip-scale 0.3	Viral genomes
ava	-e 8 -q 4 -w 3 –sig-diff 0.45 –fine-range 0.4 –min-score 40 –min-score2 75 –min-anchors 5 –min-mapq 5 –bw 5000 –max-target-gap 2500 –max-query-gap 2500	Default case

D.2. Versions

Supplementary Table S5 shows the version and the link to these corresponding versions of each tool we use in our experiments.

Table S5 Versions of each tool and library.

Tool	Version	Link to the Source Code
Rawsamble	2.1	`https://github.com/CMU-SAFARI/RawHash/releases/tag/v2.1`
Minimap2	2.24	`https://github.com/lh3/minimap2/releases/tag/v2.24`
Dorado	0.9.6 (for R9.4)	`https://github.com/nanoporetech/dorado/releases/tag/v0.9.6`
Dorado	1.0.2 (for R10.4.1)	`https://github.com/nanoporetech/dorado/releases/tag/v1.0.2`
Miniasm	0.3-r179	`https://github.com/lh3/miniasm/releases/tag/v0.3`
Rawasm	main	`https://github.com/CMU-SAFARI/rawasm`
Flye	2.9.5	`https://github.com/mikolmogorov/Flye/releases/tag/2.9.5`
HERRO	0.1	`https://github.com/lbcb-sci/herro`

Supplementary References

A. Bankevich, A. V. Bzikadze, M. Kolmogorov, D. Antipov, and P. A. Pevzner. Multiplex de bruijn graphs enable genome assembly from long, high-fidelity reads. *Nature Biotechnology*, 2022.

H. Cheng, G. T. Concepcion, X. Feng, H. Zhang, and H. Li. Haplotype-resolved de novo assembly using phased assembly graphs with hifiasm. *Nature Methods*, 2021.

B.-M. Cosma, R. Shirali HosseinZade, E. N. Jordan, P. vanLent, C. Peng, S. Pillay, and T. Abeel. Evaluating long-read de novo assembly tools for eukaryotic genomes: insights and considerations. *GigaScience*, 2022.

C. Firtina, N. Mansouri Ghiasi, J. Lindegger, G. Singh, M. B. Cavlak, H. Mao, and O. Mutlu. RawHash: enabling fast and accurate real-time analysis of raw nanopore signals for large genomes. *Bioinform.*, 2023.

C. Firtina, M. Soysal, J. Lindegger, and O. Mutlu. RawHash2: Mapping Raw Nanopore Signals Using Hash-Based Seeding and Adaptive Quantization. *Bioinform.*, 2024.

M. Kolmogorov, J. Yuan, Y. Lin, and P. A. Pevzner. Assembly of long, error-prone reads using repeat graphs. *Nature Biotechnology*, 2019.

H. Li. Minimap and miniasm: fast mapping and de novo assembly for noisy long sequences. *Bioinform.*, 2016.

H. Li. Minimap2: pairwise alignment for nucleotide sequences. *Bioinform.*, 2018.

S. Nurk, B. P. Walenz, A. Rhie, M. R. Vollger, G. A. Logsdon, R. Grothe, K. H. Miga, E. E. Eichler, A. M. Phillippy, and S. Koren. Hicanu: accurate assembly of segmental duplications, satellites, and allelic variants from high-fidelity long reads. *Genome Research*, 2020.

M. Rautiainen, S. Nurk, B. P. Walenz, G. A. Logsdon, D. Porubsky, A. Rhie, E. E. Eichler, A. M. Phillippy, and S. Koren. Telomere-to-telomere assembly of diploid chromosomes with verkko. *Nature Biotechnology*, 2023.

V. S. Shivakumar, O. Y. Ahmed, S. Kovaka, M. Zakeri, and B. Langmead. Sigmoni: classification of nanopore signal with a compressed pangenome index. *Bioinform.*, 2024.

D. Stanojevic, D. Lin, P. Florez De Sessions, and M. Sikic. Telomere-to-telomere phased genome assembly using error-corrected simplex nanopore reads. *arXiv*, 2024.

J. Sun, R. Li, C. Chen, J. D. Sigwart, and K. M. Kocot. Benchmarking oxford nanopore read assemblers for high-quality molluscan genomes. *Philosophical Transactions of the Royal Society*, Apr. 2021.

R. R. Wick and K. E. Holt. Benchmarking of long-read assemblers for prokaryote whole genome sequencing. *F1000Research*, 2021.

W. Yu, H. Luo, J. Yang, S. Zhang, H. Jiang, X. Zhao, X. Hui, D. Sun, L. Li, X.-q. Wei, S. Lonardi, and W. Pan. Comprehensive assessment of 11 de novo hifi assemblers on complex eukaryotic genomes and metagenomes. *Genome Research*, 2024.

H. Zhang, H. Li, C. Jain, H. Cheng, K. F. Au, H. Li, and S. Aluru. Real-time mapping of nanopore raw signals. *Bioinformatics*, 37 (Supplement_1):i477–i483, July 2021. ISSN 1367-4803.
